# Inorganic phosphate accelerates cardiac myofilament relaxation in response to lengthening

**DOI:** 10.3389/fphys.2022.980662

**Published:** 2022-09-12

**Authors:** Jane I. Wakefield, Stephen P. Bell, Bradley M. Palmer

**Affiliations:** ^1^ Department of Biology, University of Vermont, Burlington, VT, United States; ^2^ Department of Medicine, Larner College of Medicine, University of Vermont, Burlington, VT, United States; ^3^ Department of Molecular Physiology and Biophysics, Larner College of Medicine, University of Vermont, Burlington, VT, United States

**Keywords:** cardiac, myosin, crossbridge, phosphate, diastole

## Abstract

Myocardial relaxation in late systole is enhanced by increasing velocities of lengthening. Given that inorganic phosphate (Pi) can rebind to the force-producing myosin enzyme prior to MgADP release and hasten crossbridge detachment, we hypothesized that myocardial relaxation in late systole would be further enhanced by lengthening in the presence of Pi. Wistar rat left ventricular papillary muscles were attached to platinum clips, placed between a force transducer and a length motor at room temperature, and bathed in Krebs solution with 1.8 mM Ca^2+^ and varying Pi of 0, 1, 2, and 5 mM. Tension transients were elicited by electrical stimulation at 1 Hz. Peak tension was significantly enhanced by Pi: 0.593 ± 0.088 mN mm^−2^ at 0 mM Pi and 0.817 ± 0.159 mN mm^−2^ at 5 mM Pi (mean ± SEM, *p* < 0.01 by ANCOVA). All temporal characteristics of the force transient were significantly shortened with increasing Pi, e.g., time-to-50% recovery was shortened from 305 ± 14 ms at 0 mM Pi to 256 ± 10 ms at 5 mM Pi (*p* < 0.01). A 1% lengthening stretch with varying duration of 10–200 ms was applied at time-to-50% recovery during the descending phase of the force transient. Matching lengthening stretches were also applied when the muscle was not stimulated, thus providing a control for the passive viscoelastic response. After subtracting the passive from the active force response, the resulting myofilament response demonstrated features of faster myofilament relaxation in response to the stretch. For example, time-to-70% relaxation with 100 ms lengthening duration was shortened by 8.8 ± 6.8 ms at 0 Pi, 19.6 ± 4.8* ms at 1 mM Pi, 31.0 ± 5.6* ms at 2 Pi, and 25.6 ± 5.3* ms at 5 mM Pi (*p < 0.01 compared to no change). Using skinned myocardium, half maximally calcium-activated myofilaments underwent a 1% quick stretch, and the tension response was subjected to analysis for sensitivity of myosin detachment rate to stretch, *g*_1_, at various Pi concentrations. The parameter *g*_1_ was enhanced from 15.39 ± 0.35 at 0 Pi to 22.74 ± 1.31 s^−1^/nm at 8 Pi (*p* < 0.01). Our findings suggest that increasing Pi at the myofilaments enhances lengthening-induced relaxation by elevating the sensitivity of myosin crossbridge detachment due to lengthening and thus speed the transition from late-systole to early-diastole.

## Introduction

The duration of the isovolumic relaxation phase of the cardiac cycle depends upon the rate at which LV contractile force subsides during the transition from late-systole to early-diastole. The decline of intracellular Ca^2+^ is an important contributor to this transition, because Ca^2+^ decline deactivates the thin filament and inhibits the formation of new force-producing myosin crossbridges (Bers, 2002). The detachment rate of myosin crossbridges is another important component. A faster rate of crossbridge detachment translates to faster LV relaxation (Kass et al., 2004). Under normal physiological conditions, which includes saturating MgATP concentrations, further enhancing of crossbridge detachment rate can be achieved via post-translational modification of key myofilament proteins, such as by phosphorylation of myosin binding protein-C (Stelzer et al., 2006).

Using force-clamp experiments, Chung et al. (2017) showed that stretching the myocardium at end-systole also enhances the rate of LV myocardial relaxation. This enhancement of LV relaxation by stretch was greatest with the fastest rates of stretch, which suggests that myosin crossbridge detachment is sensitive to stretch. This and similar findings at the whole heart level (Zile et al., 2015) are relevant to the clinical treatment of heart failure with preserved ejected fraction (Dunlay et al., 2017), as they support the idea that myocardial stretch can enhance LV relaxation during the transition from late-systole to early-diastole.

Knowing that Pi can facilitate myosin detachment due to stretch at the molecular level (Dantzig et al., 1992; Smith and Geeves, 1995), we hypothesized that stretch-induced myocardial relaxation would be enhanced by increasing Pi concentrations. In the current study, we examined isometric excitable myocardium over various extracellular Pi concentrations and imposed a lengthening stretch in late-systole to demonstrate the effects of Pi in hastening the transition to early-diastole.

## Methods

### Solutions

Chemicals and reagents were obtained from Sigma-Aldrich Corp. (St. Louis, MO) unless otherwise noted. Krebs-Ringer Pi-free bathing solution concentrations (mmol/L) were 115 NaCl, 4.5 KCl, 1.8 CaCl_2_, 1 MgCl_2_, 25 NaHCO_3_, 10 glucose, pH 7.35–7.40. Pi-containing bathing solutions (1, 2, and 5 mM), which cover the physiological range of 0.8–1.3 mM extracellular Pi (Manghat et al., 2014; Jacquillet and Unwin, 2019), were produced by replacing the same concentrations of NaCl with NaH_2_PO_4_. Propranolol was included in all bathing solutions at 1 μM.

Relaxing solution: pCa 8.0, 5 EGTA, 5 MgATP, 1 Mg^2+^, 35 phosphocreatine (PCr), 300 U/ml creatine kinase (CK), ionic strength 200, pH 7.0; activating solution: same as relaxing with pCa 4.0 and 0–8 mM Pi; skinning solution: same as relaxing without CK, with 1% Triton-X100 wt/vol, 30 mM 2,3-butanedione 2-monoxime (BDM), 10 uL/ml E-64, 1.25 uL/ml Phenylmethylsulfonyl fluoride (PMSF), 1 tablet/10 ml PhosSTOP phosphatase inhibitor cocktail and 50% glycerol wt/vol; storage solution: same as skinning without Triton-X100. The pH of all solutions was adjusted at the temperature used.

### Animal model

All procedures were reviewed and approved by the Institutional Animal Care and Use Committee of The University of Vermont Larner College of Medicine and complied with the *Guide for the Use and Care of Laboratory Animals* published by the National Institutes of Health. Adult female Wistar rats aged 4–5 months were acquired from Charles River. Animals were anesthetized with 2%–4% isoflurane and hearts removed.

### Excitable papillary muscle

Hearts were submerged immediately into a Ca^2+^-free Krebs-Ringer + 30 mM BDM solution bubbled with 95%O_2_-5%CO_2_ to pH 7.35–7.40 and maintained at 4°C. The large blood vessels, atria, and valves were removed from the base of the ventricles. The right ventricle free wall was removed, and an incision made from the base to the apex along the interventricular septum, therefore exposing the anterior and posterior papillary muscles. Both papillary muscles were excised, trimmed to 0.8–1.4 mm diameter, placed between two platinum omega-shaped clips, and tied with 6-O suture (Selby et al., 2011; Runte et al., 2017). Relative fiber dimensions of top width and side width were measured for later calculation of cross-sectional area.

Clipped muscle was placed between a force transducer (TR6S, Myotronic, Heidelberg, Germany) and voice coil length motor with ±2.5 mm travel (V-522, Physik Instrumente, Auburn, MA) part of a small intact muscle chamber (IonOptix, Westwood, MA) maintained at room temperature. A biphasic electrical stimulus of 1.5 × threshold voltage and 5 ms pulse width was passed through the muscle *via* the platinum hooks on which the clips rested.

Muscles were slowly stretched to an optimal length that achieved maximal peak force. Muscle length (ML) was then measured between sutures and cross-sectional area calculated from the measured major diameter and inferred minor diameter. Force (F) transients were recorded in response to electrical stimuli and were stabilized for 5 min prior to characterizing the transient with peak force, +dF/dt_max_, −dF/dt_min_, time to peak, time to 50% peak, and times to 40%, 50%, 60%, 70% and 80% recovery to baseline. Tension (P) was calculated by normalizing force to cross-sectional area.

Length changes were applied at time to 50% recovery and followed the shape of a raised cosine: 0.5 × Amp × [1 − cos (πt/T)], where Amp = ending extent of stretch calculated as 1% muscle length, t = time, and T = lengthening period ranging from 10 to 200 ms. The stretch was held for 240–50 ms, and the muscle then shortened over 50 ms back to the original muscle length. The lengthening protocol was also applied when the electrical stimulus was absent, which served as a measure of the force response of the passive components, i.e., excluding myofilaments. The force response to the stretch attributable to active myofilaments was calculated as active response minus passive response.

### Demembranated papillary muscle

A portion of papillary muscle not used in excitable strip experiments were demembranated overnight at 4°C in skinning solution then stored at −20°C in storage solution. Strips were trimmed to 150–220 μm diameter before attaching aluminum T-clips and then mounted between a piezoelectric motor (P841.60, Physik Instrumente, Auburn, MA) and a strain gauge (AE801, Kronex, Walnut Creek, CA), lowered into a 30 μl droplet of relaxing solution maintained at room temperature, and stretched to 2.2 μm sarcomere length (IonOptix, Westwood, MA).

Muscles were maximally activated with pCa 5 and then approximately half-maximally activated with pCa 5.75. Force responses were recorded after step length changes of 1% resting muscle length (ML) during exposure to 0, 2, 4, and 8 mM Pi, which covers the physiological range of cytosolic Pi 4–8 mM (Tian et al., 1997; Valkovič et al., 2019). The stress response at pCa 8, *P*_8_(*t*), was subtracted from the stress response at pCa 5.75, *P*_5.75_(*t*), and the result was used as the response of the myofilaments. Analysis of this myofilament-dependent stress response, *P*_*m*_(*t*), resulted in values for peak tension of phase 1 (P_1_), minimum tension of phase 2 (P_2_), maximum redeveloped tension of phase 3 (P_3_), and rates of force release (k_rel_) and force redevelopment (k_redev_) (Palmer et al., 2020).

### Modeling stretch-induced relaxation

The myofilament-dependent stress response, *P*_*m*_(*t*), was further subjected to a non-linear, least-squares fit to the following equation, which describes the mechanical consequences of enhanced myosin crossbridge detachment in a two-state model of myosin kinetics when the half sarcomere undergoes strain, *ε*, due to a quick stretch (Palmer et al., 2020):

$$P_{m}\left(t\right)=\varepsilon G_{P}t^{-k_{p}}-\underset{R}{\underset{︸}{\varepsilon L_{hs}\left[\frac{N_{hs}F_{uni}}{CSA}\right]\frac{g_{1}{\bar{A}}_{0}}{f_{0}}}}\left(e^{-g_{0}t}-e^{-\left(f_{0}+g_{0}\right)t}\right)+\underset{D}{\underset{︸}{\varepsilon L_{hs}\left[\frac{N_{hs}k_{stiff}}{CSA}\right]{\bar{A}}_{0}}}e^{-g_{o}t}$$ (1)

The meanings of the terms and parameters are comprehensively explained in Palmer et al. (2020). Briefly, the first term of Eq. 1 refers to the passive power-law relaxation that characterizes the slow decline in stress that occurs after the active force generating mechanisms have completed their response. The second term describes the transient drop in isometric force that results from the temporarily enhanced detachment rate caused by the stretch. Because this second term is modeled to arise from the reversal of the myosin power-stroke caused by stretch, we will refer to the magnitude of this term as *R* for reversal. The third term describes the transient elevation in stress that results from those crossbridges that are initially stretched by the length change but eventually detach with a rate constant of *g*_0_. We will refer to the magnitude of this term as *D* for drag.

The parameters in Eq. 1 are defined as follows: *G*_*p*_ and *k*_*p*_ refer to the magnitude and time characteristic, respectively, of the passive power-law relaxation, *L*_*hs*_ = length of half sarcomere such that *εL*_*hs*_ is the length change in nm applied to the myosin crossbridges, *N*_*hs*_ = total number of available myosin crossbridges within a half sarcomere of the muscle under investigation, *F*_*uni*_ = unitary force generated by a single crossbridge in pN, *k*_*stiff*_ = stiffness of a single attached crossbridge in pN/nm, *CSA* = cross-sectional area in mm^2^, *g*_1_ = the change in the myosin crossbridge detachment rate that occurs with strain on the crossbridge in units of s^−1^/nm, ${\bar{A}}_{0}$ = stead-state fraction of myosin crossbridges attached, *f*_0_ = rate of myosin crossbridge attachment, and *g*_0_ = rate of myosin crossbridge detachment under steady-state conditions. It should be noted that, as written, the conventional rates k_rel_ and k_redev_ are related to the attachment and detachment rates of a model presented in Eq. 1 as k_rel_ = (*f*_0_ + *g*_0_) and k_redev_ = *g*_0_.

The constant *g*_1_ refers to the enhancement of myosin crossbridge detachment rate caused by stretch and therefore was used as an index of stretch-induced relaxation. The magnitudes of the second and third terms in Eq. 1, *R* and *D*, were used to estimate *g*_1_ as follows:

$$g_{1}=\frac{R}{D}f_{0}\left[\frac{k_{stiff}}{F_{uni}}\right]$$ (2)

The values for *R*, *D*, and *f*_*0*_ were estimated from the fit of Eq. 1 to the data. The unitary force *F*_*uni*_ is related to the crossbridge stiffness *k*_*stiff*_ by the myosin power stroke unitary displacement *d*_*uni*_ as *F*_*uni*_ = *k*_*stiff*_ *d*_*uni*_, where *d*_*uni*_ is ∼10 nm (Tyska and Warshaw, 2002). We estimated the ratio *k*_*stiff*_/*F*_*uni*_ with a constant value of 0.1 nm^−1^.

### Statistical analysis

Statistical analyses to demonstrate the Pi-sensitivity of parameters describing stimulated isometric force transients was performed using ANCOVA with the Pi condition used as the covariate. Differences in isometric force parameters prior to and post the stretch protocol were examined by paired *t*-test.

Analysis to demonstrate duration-sensitivity of parameters affected by the stretch protocol was performed using ANCOVA with 20 transition durations (10–200 ms duration) and four Pi conditions (0, 1, 2, and 5 mM Pi) used as covariates. The effects of transition duration were demonstrated by duration main effects and of extracellular Pi by Pi main effects, and the differential Pi-sensitivity of transition durations were tested with Duration × Pi interactions. Statistical analyses to demonstrate the Pi-sensitivity of parameters describing the force response to a quick stretch was performed by ANCOVA with four Pi conditions (0, 2, 4, and 8 mM Pi) used as covariates. Statistical significance was reported at *p* < 0.05 and *p* < 0.01 levels.

## Results

### Isometric force transients

**FIGURE 1:**
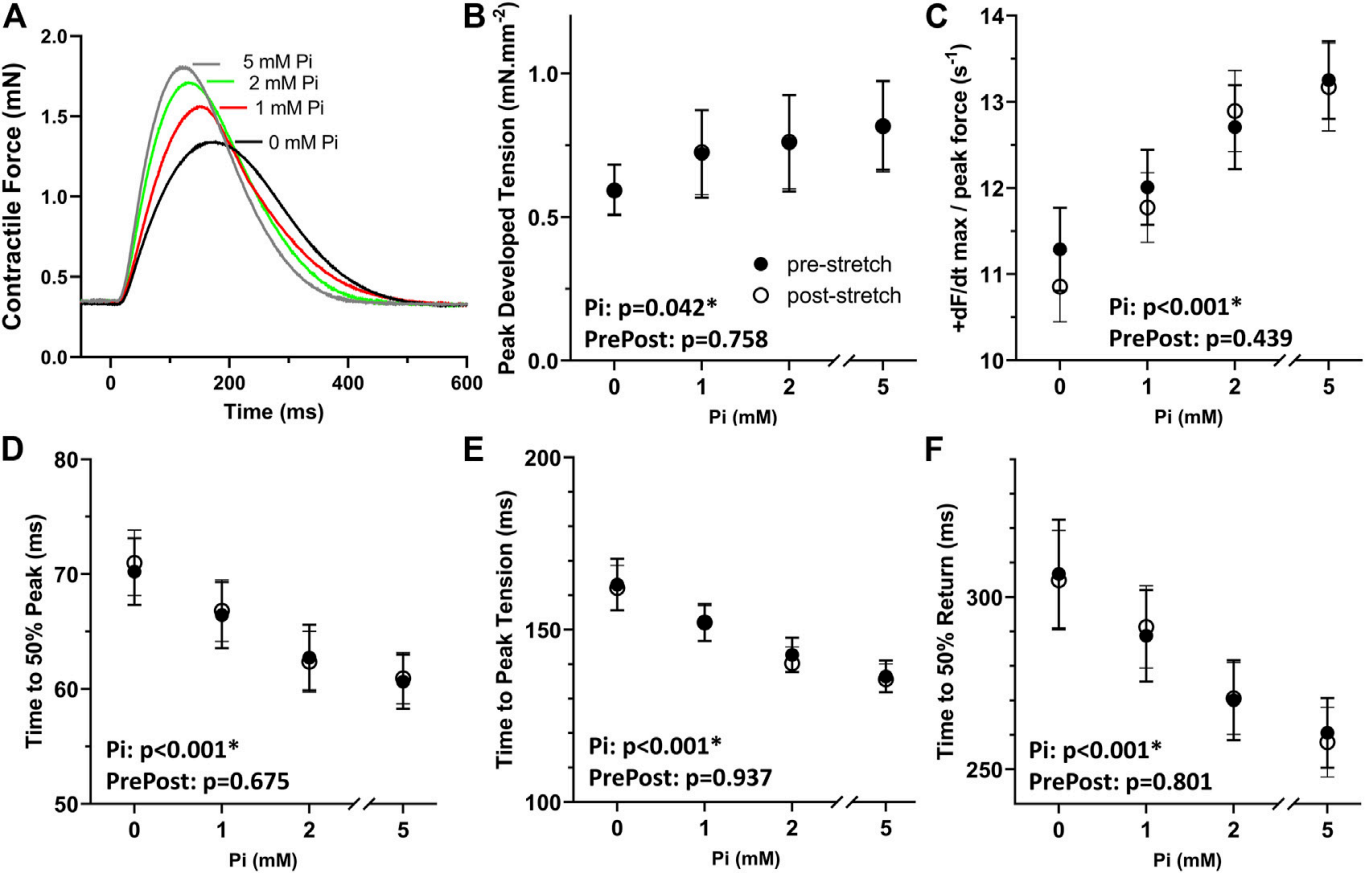
Effects of extracellular Pi on isometric myocardial force transients. **(A)** Examples of isometric force transients recorded at 0, 1, 2, and 5 mM extracellular Pi demonstrate a Pi-induced enhancement of systolic and diastolic function. **(B)** Developed peak tension was enhanced with increasing Pi. **(C)** Maximum rate of force increase normalized to peak force was also enhanced with increasing Pi. **(D,E)** Temporal characteristics of systolic function, time to 50% peak and time to peak, were shortened significantly by increasing Pi. **(F)** Time to 50% return was significantly shortened by increasing Pi thus indicating Pi-enhanced diastolic function. *n* = 8 rat papillary muscles.

Increasing extracellular Pi led to enhanced systolic and diastolic function in isometric rat papillary muscle (Figure 1A). Pi-enhanced systolic function was observed as higher developed peak tension, faster rate of contraction normalized to developed peak, shorter time to 50% peak, and shorter time to peak (Figures 1B–E) detected by ANCOVA. Enhanced diastolic function was reflected in a shorter time to peak and shorter time to 50% return (Figures 1E,F), which were significantly affected by Pi.

The results in Figures 1B–F also demonstrate that the stretching protocol, which is described in the next section, did not appreciably affect the isometric force transients. Transient parameters recorded after the stretch, i.e., post-stretch, were not different compared to pre-stretch parameters as demonstrated by non-significant ANCOVA.

### Stretch protocol

**FIGURE 2:**
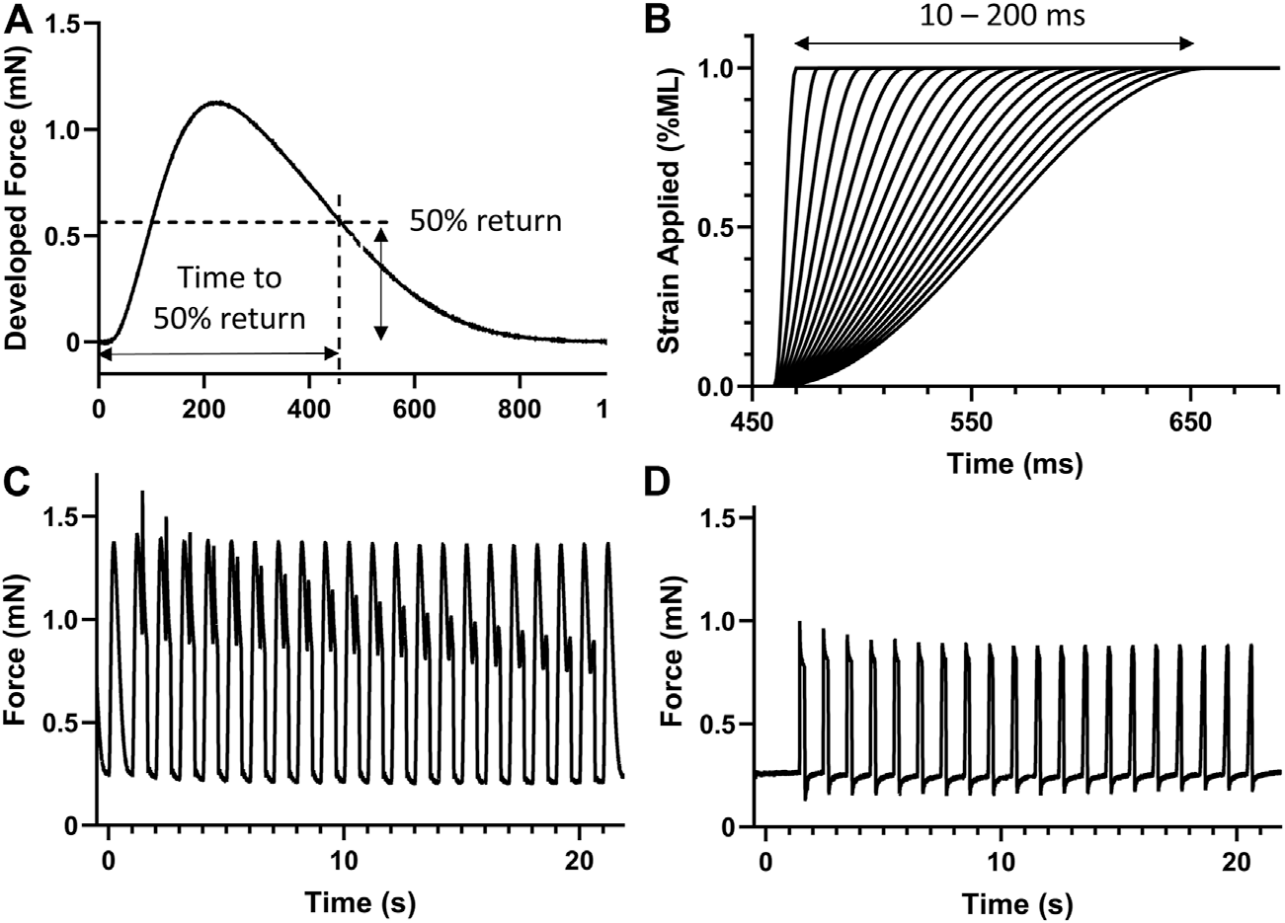
Stretch protocol. **(A)** The time to 50% return was noted for the isometric force transient. **(B)** A series of 20 stretches from 10 to 200 ms in duration were generated to apply a 1% strain on the muscle at the time of 50% return, i.e., 461 ms in this case. **(C)** Example stretch protocol applied to stimulated isometric force transients. **(D)** The same stretch protocol was also applied without stimulation to record the expected force response of passive components. *n* = 8 rat papillary muscles.

After establishing at least 5 min steady state contractile dynamics at each Pi condition, the time to 50% return was noted (Figure 2A). A series of 20 stretches from 10 to 200 ms in duration was then applied at the time to 50% return (Figure 2B). The strain of each stress was 1% muscle length and followed a smooth half-cosine transition. The 20 stretched transients, including a pre- and post-stretch transient, are shown in Figure 2C. The same stretch protocol was applied without stimulation to provide the response of the passive components of the muscle (Figure 2D).

**FIGURE 3:**
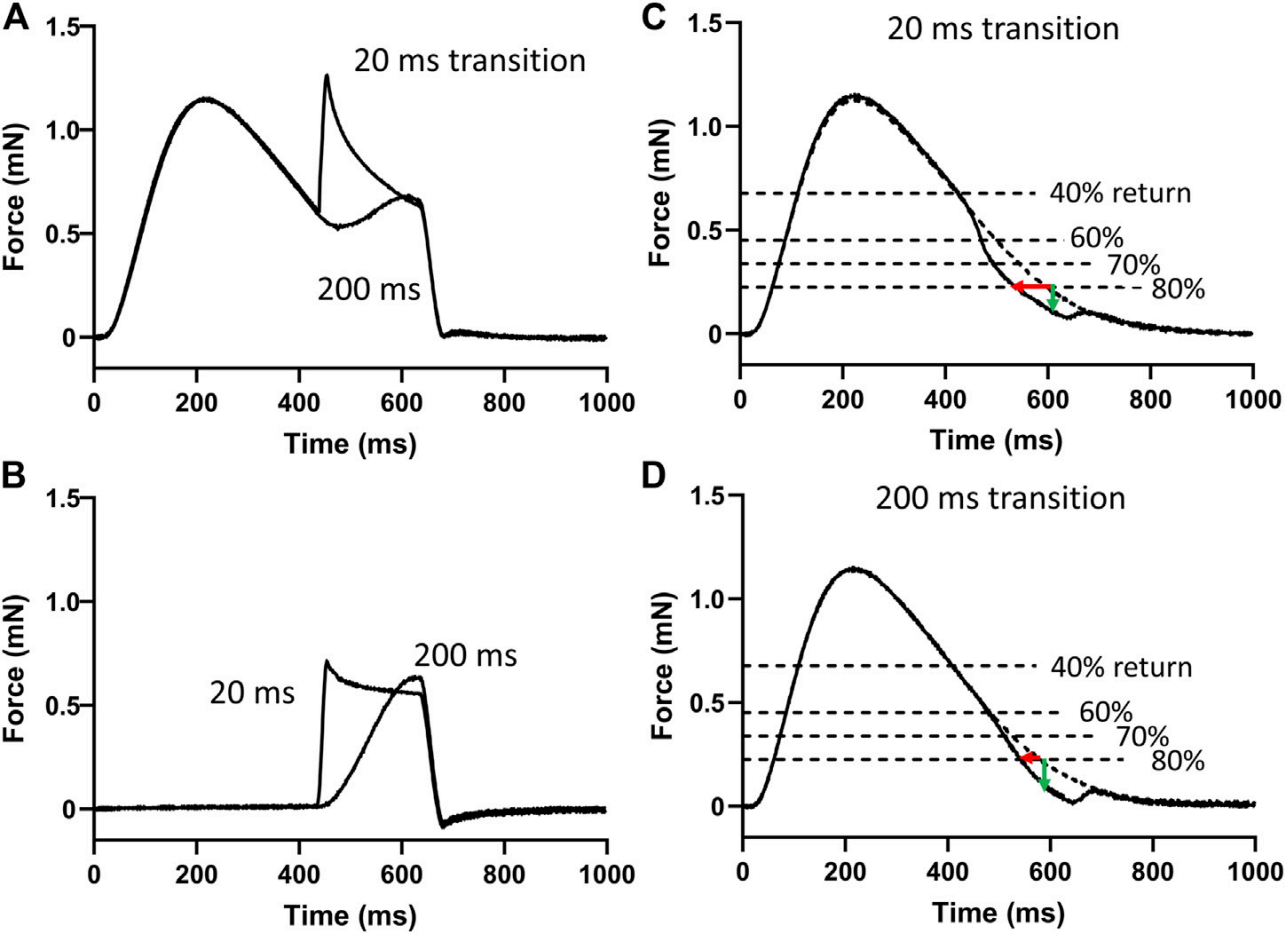
Analysis of myofilament response to stretch. **(A)** Two of the 20 force responses recorded during stimulation are overlayed here and represent the force responses of the myofilaments and the passive components during the 20 and 200 ms stretches. **(B)** The corresponding two force responses recorded without stimulation represent the passive components. These responses without stimulation were subtracted from those with stimulation to provide the myofilament response to stretch. **(C)** An example myofilament response to a 20 ms duration stretch to 1% muscle length. Times to 40%, 60%, 70%, and 80% return were detected. The effects of stretch on shortening of these times (red arrow) and on the loss of force at these times (green arrow) relative to the pre-stretch transient were detected. **(D)** An example myofilament response to a 200 ms duration stretch to 1% muscle length.

Two example force transients recorded with 20 and 200 ms stretch responses are shown overlayed in Figure 3A. Responses such as these was considered due to the force-producing myofilaments plus the passive components of the myocardium. The corresponding stretch responses without stimulated transients are shown in Figure 3B and was considered due to passive components only. The passive responses were subtracted from the myofilament plus passive responses to produce the myofilament only responses. Figures 3C,D illustrate examples of the responses to 20 and 200 ms duration stretches, respectively.

The times to 40%, 60%, 70%, and 80% returns in these myofilament-only responses were detected and compared to the same time points recorded in the pre-stretch isometric force transient (dashed line in Figures 3C,D). The differences in these time points were used to demonstrate the effects of stretch and extracellular Pi on enhancing relaxation during the transition from late-systole to early-diastole.

### Effects of Pi on stretch response in late-systole

**FIGURE 4:**
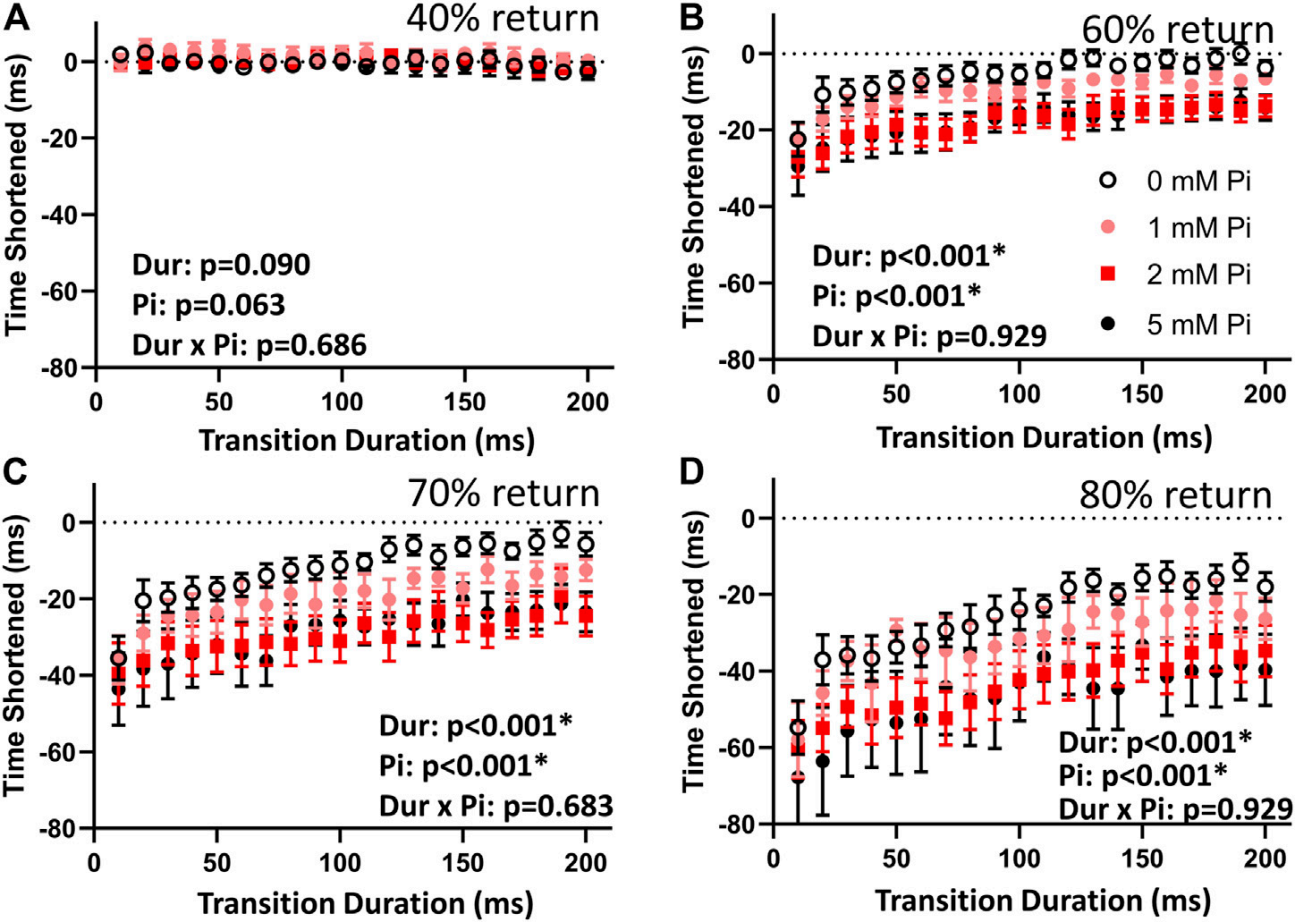
Effects of stretch on times to return. **(A)** The time to 40% return was not affected by lengthening imposed at time to 50% return. This result indicates that the stimulated transients were repeatable and comparable throughout the lengthening protocol by this measure. **(B)** When no extracellular Pi was available (0 mM Pi), the time to 60% return was abbreviated by those 1% stretches imposed over less than 110 ms. Faster stretches resulted in shorter times to 60% return, as indicated by the highly significant duration (Dur) main effect. This abbreviation was amplified with increasing extracellular Pi as indicated by a highly significant Pi main effect. **(C)** With no extracellular Pi, the time to 70% return was abbreviated by stretches imposed over less than 170 ms. This effect was amplified by extracellular Pi. **(D)** The time to 80% return was also abbreviated by all stretches under all conditions but was especially abbreviated by faster stretches and increasing extracellular Pi.

The difference in times to 40% return were not affected by stretch or by Pi (Figure 4A). This result demonstrates that the stimulated isometric force transients up to the point of time to 50% return were consistent in their dynamics.

The times to 60%, 70%, and 80% return (Figures 4B–D) for the myofilaments-only transients were shortened by stretch. Shortening was greatest with the fastest stretches as indicated by a significant duration main effect (*p* < 0.01) for these variables. Furthermore, increasing extracellular Pi amplified the effects of stretch as indicated by the significant Pi main effect (*p* < 0.01). There was no significant duration × Pi interaction.

**FIGURE 5:**
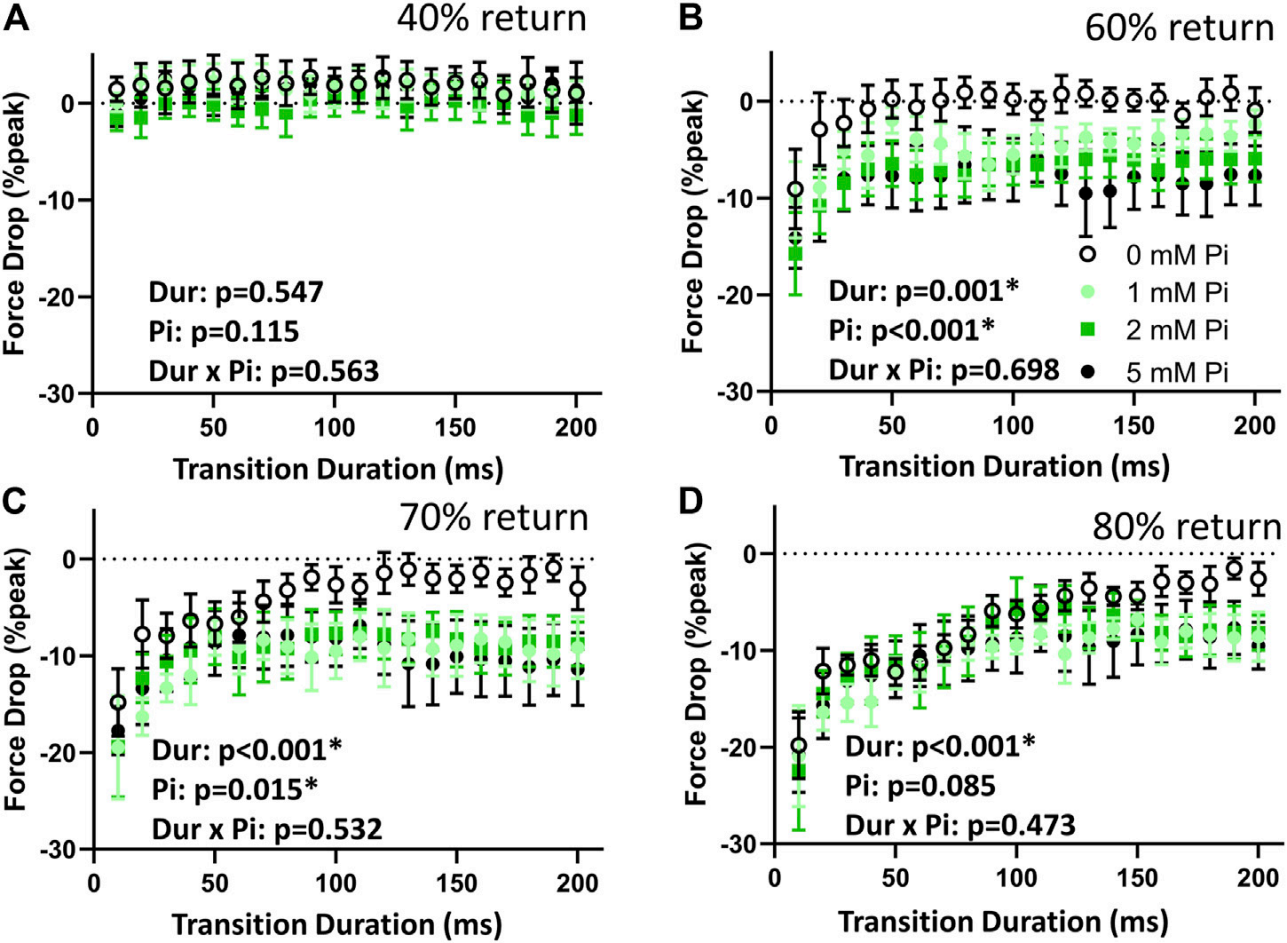
Effects of stretch on force drop. **(A)** The force drop at the 40% return time point was not affected by the lengthening protocol at any Pi concentration. The stimulated transients were therefore not affected by lengthening by this measure. **(B)** When no extracellular Pi (0 mM Pi) was available, the drop in force at 60% return timepoint was significant with the 10 ms stretch but was not significant for the more prolonged stretches. This dependence on duration (Dur) was highly significant. The drop in force at the 60% return timepoint was significantly enhanced by Pi. **(C)** With no extracellular Pi, force drop at the 70% return timepoint was significant for stretches of 80 ms duration or shorter. Force drop was significant at all stretch durations when extracellular Pi was available. **(D)** Force drop at the 80% return timepoint was significant for stretches of 170 ms or shorter and for all durations when extracellular Pi was available.

The drop in force at time to 40% return was not affected by stretch or by Pi (Figure 5A) demonstrating again the consistency in the transient characteristics throughout the stretch protocol. The drop in force at times to 60%, 70%, and 80% return (Figure 5B–D) were sensitive to stretch duration (*p* < 0.01) and to Pi (*p* < 0.01 for 60% and *p* < 0.05 for 70%, but not significant for 80%). There were no significant duration × Pi interactions for the measured drop in force at these time points.

### Effects of Pi on stretch response in skinned myocardium

**FIGURE 6:**
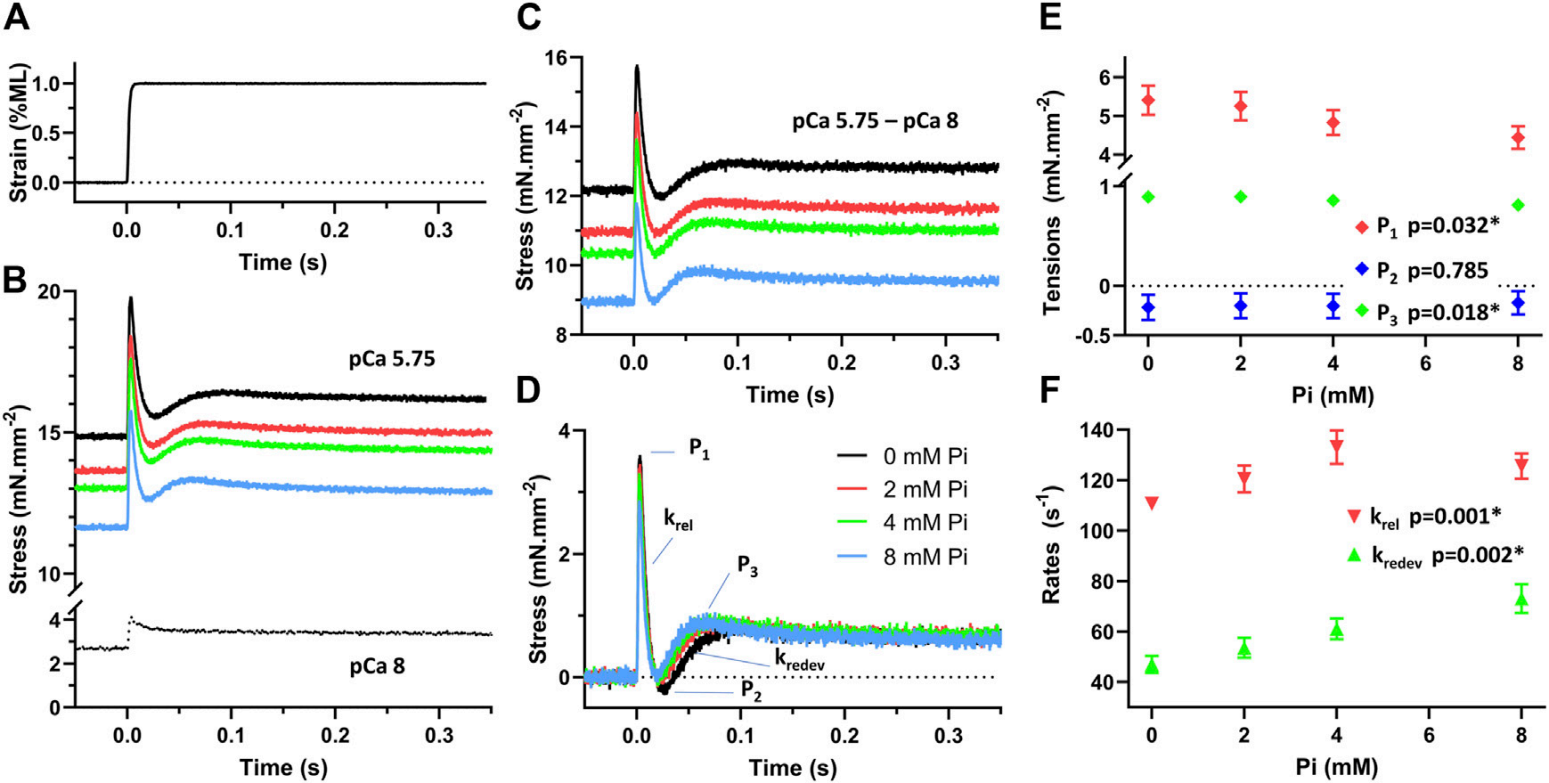
Stress response to stretch of demembranated myocardium. **(A)** Muscle was stretched with a quick stretch of 1% muscle length (ML). **(B)** The stress response was recorded under relaxed conditions, pCa 8, and at approximately half-activation, pCa 5.75, with 0, 2, 4, and 8 mM Pi. **(C)** The activated response attributable to the force-producing myofilaments was determined by subtracting the pCa 8 response from the pCa 5.75 responses. **(D)** The stretch response of the myofilaments above the myofilament isometric stress was then determined. This response represents the stress change due to the myofilaments after a quick stretch. **(E)** Peak tension (P_1_), lowest tension at nadir (P_2_), and recovered tension (P_3_) were recorded in response to Pi concentration. P_1_ and P_3_ were significantly reduced with increasing Pi, *p* < 0.05. P_2_ was not found sensitive to Pi. **(F)** Rates of tension release (k_rel_) and redevelopment (k_redev_) were significantly enhanced with increasing Pi as indicated by *p* < 0.01.

To demonstrate the Pi-dependent sensitivity of the force-producing myofilaments in response to stretch, we applied a 1% quick stretch (Figure 6A) to approximately half-activated myofilaments at pCa 5.75. Responses were recorded during exposure to 0, 2, 4, and 8 mM Pi (Figure 6B). The response under relaxed conditions at pCa 8 was subtracted from the responses at pCa 5.75 to produce the stretch response attributable to the myofilaments (Figure 6C). The stress response relative to the isometric stress prior to stretch (Figure 6D) represented the Pi-sensitivity of the myofilament-dependent stress response to quick stretch.

The characteristic stresses (P_1_, P_2_, and P_3_) and rates of release and redevelopment (k_rel_ and k_redev_) are presented in Figures 6E,F, respectively. Increasing concentrations of Pi induced a reduction in P_1_ and P_3_ (*p* < 0.05) which suggests that fewer myosin crossbridges are formed in the presence of Pi. Increasing Pi also led to enhancement of the rates k_rel_ and k_redev_ (*p* < 0.01 for both rate parameters) as reported previously by others (Straight et al., 2019).

### Modeling stretch response in skinned myocardium

The skinned myocardial response to quick stretch permitted use of previous modeling efforts to interpret the effects of Pi on the stretch-induced detachment rate of myosin. The parameter *g*_1_ represents the change in myosin detachment rate per change in length experienced by the myosin crossbridge while attached. Through the parameters calculated by fitting Eq. 1 to the recorded stress responses, we expected to estimate *g*_1_ using Eq. 2 and then test for its sensitivity to Pi.

**FIGURE 7:**
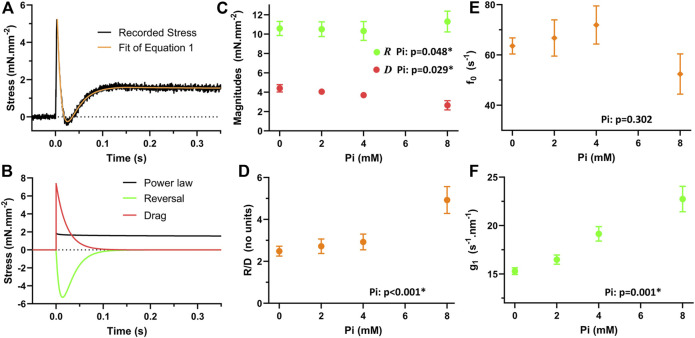
Modeled response of myofilaments to quick stretch. **(A)** Eq. 1 was fit to the myofilament response to a quick stretch. **(B)** The three terms of Eq. 1 demonstrate how each term contributes to the response. Power-law relaxation term constitutes the resultant “stretch activated” stress. The reversal term is responsible for the transient drop in the stress. The drag term constitutes the initial brief rise in stress after the stretch. **(C)** The magnitudes of the reversal and drag terms were sensitive to Pi. The magnitude of reversal, *R*, was significantly enhanced with increasing Pi, *p* < 0.05. The magnitude of drag, *D*, was significantly reduced with increasing Pi, *p* < 0.05. **(D)** The ratio of *R/D* was significantly enhanced with increasing Pi, *p* < 0.01. **(E)** According to Eq. 1, the attachment rate, *f*_*0*_, can be determined by subtracting the k_redev_, equivalent to *g*_*0*_ in the model, from k_rel_, equivalent to *f*_*0*_ + *g*_*0*_ in the model. This attachment rate was not found to be significantly influenced by Pi. **(F)** The sensitivity of the detachment rate to stretch, *g*_*1*_ in the model and calculated using Eq. 2, was found to be significantly enhanced with increasing Pi, *p* < 0.01.

An example myofilament-dependent stress response is presented in Figure 7A, and the fit of Eq. 1 to this response demonstrated no more than 3% error relative to the stress at 1 s after the stretch (Palmer et al., 2020). The three terms that make up the model are plotted in Figure 7B. The power-law relaxation term represents the long term elevation in stress that persists after the stretch. The reversal term describes the transient reduction in myofilament-dependent stress and therefore the transient drop in the number of myosin crossbridges producing force. And the drag term is responsible for the initial peak stress immediately after the stretch.

The magnitude for the reversal term, *R*, was statistically significantly elevated with increasing Pi (Figure 7C, *p* < 0.05), but the relative change was only on the order of 5%–10% across the range of Pi concentrations investigated. The magnitude of the drag term, *D*, was significantly reduced with increasing Pi (Figure 7C, *p* < 0.05). The ratio R/D, which is required to apply in Eq. 2, was found to be highly significantly enhanced with Pi (Figure 7D, *p* < 0.01).

The myosin attachment rate, *f*_0_, is also necessary for the application of Eq. 2 and calculation of *g*_1_. The value of *f*_0_ was calculated by subtracting k_redev_ from k_rel_, which according to the model represent (*f*_0_ *+ g*_0_) and *g*_0_, respectively (Palmer et al., 2020). This attachment rate was not found to be significantly affected by Pi (Figure 7E, *p* > 0.05).

The resulting values for *g*_1_ were found to be significantly enhanced with increasing Pi concentrations (Figure 7F, *p* < 0.01). This result suggests that Pi enhances the increase in myosin detachment rate that occurs with stretch imposed on the crossbridge.

## Discussion

This study demonstrated the significance of Pi in stretch-dependent relaxation function of excitable rat myocardium. We confirmed that increasing extracellular Pi enhanced both systolic and diastolic function as reported previously at the whole heart level (Onwochei, 1995; Onwochei et al., 1998). We also found that the rate of relaxation during the transition from late-systole to early-diastole is enhanced by stretch and more so with the higher rates of stretch imposed upon the myocardium, which is consistent with the findings of Chung et al. (2017). The novel result of this study was the demonstration that this increase in relaxation rate by stretch is further enhanced by the presence of Pi. Using skinned myocardium, we were able to demonstrate that Pi enhances the stretch sensitivity of the myosin crossbridge detachment rate, thus providing the underlying mechanism by which stretch enhances relaxation rate at the whole heart (Zile et al., 2015) and myocardial levels (Chung et al., 2017, present work).

The rate of myosin detachment is typically dictated by the rate of MgADP release, which occurs as the myosin crossbridge produces force (Tyska and Warshaw, 2002; Nyitrai and Geeves, 2004). Importantly, the rate of MgADP release is slowed by a resistive load, i.e., an external force that resists the force produce by the crossbridge, which would be experienced by any force-producing myosin being pulled back as would occur during muscle relengthening (Smith and Geeves, 1995; Nyitrai and Geeves, 2004; Veigel et al., 2005; Kad et al., 2007; Liu et al., 2018). If the rate of MgADP release is slowed by lengthening, then the myosin crossbridge lifetime would be prolonged and relaxation would be hindered. The results of the current study and of others (Zile et al., 2015; Chung et al., 2017) do not reflect a slowing of MgADP release from the myosin crossbridge due to sarcomere lengthening. It appears that some other mechanism or some other set of enzymatic steps independent of MgADP release hasten myosin detachment when the sarcomere is lengthened.

One alternative enzymatic pathway to myosin crossbridge force cessation is *via* rebinding of Pi thus prompting reversal of the myosin power stroke (Dantzig et al., 1992; Smith and Geeves, 1995; Woody et al., 2019) or crossbridge detachment (Debold et al., 2013). Cytosolic Pi concentration is expected in the range 2–8 mM in cardiac muscle (Tian et al., 1997; Valkovič et al., 2019). This range of Pi concentrations is known to enhance myosin kinetics and the frequency characteristics of dynamic stiffness of demembranated cardiac muscle (Kawai et al., 2016) as well as skeletal muscle (Ranatunga, 1999). The possibility of reversal of the myosin power stroke is consistent with the findings of reduced Pi release rate during muscle lengthening (Mansfield et al., 2012) in demembranated cardiac muscle. The specific mechanism affected by Pi in excitable myocardial contractile-relaxation function is difficult to study directly, in part because monitoring cytosolic Pi while also measuring myocardial function is not yet technically feasible.

Using demembranated myocardium and applying previous modeling results (Palmer et al., 2020), we were able to demonstrate that Pi enhanced the sensitivity of the rate of myosin crossbridge detachment to stretch of the crossbridge as reflected in the parameter *g*_1_. We were able to estimate *g*_1_ after fitting Eq. 1 to the recorded stress response to a quick stretch and applying the resulting parameter values according to Eq. 2. The current set of results lends some credibility to the model as the expected enhancement of myosin detachment rate by Pi was demonstrated for the relevant model parameters, i.e., *R*, *D*, and *g*_1_.

### Limitations

While the demembranated myocardium preparation provided some insights into the effects of Pi on cardiac myosin, it did not mimic the results found with excitable muscle. Specifically, the stretch response in the activated demembranated myocardium was significantly greater in magnitude than that of the relaxed demembranated myocardium. The stretch response of the myofilaments was therefore positively valued even if reduced with increasing Pi. In the excitable muscle, on the other hand, stretches applied during the stimulated tension transient were lower than those under relaxed conditions. The stretch response of the myofilaments was negatively valued.

One difference between the two preparations is the activation status of the thin filament. The thin filament is deactivated during the imposed stretch in the excitable myocardium and activated during stretch in the demembranated myocardium. It appears that the enhancement of relaxation function observed with stretch and further enhanced by Pi reflects the consequences of Pi on myosin detachment and most evident when attachment of myosin crossbridges is inhibited.

Another difference between the two preparations is the presence of Ca^2+^-regulatory mechanisms in the excitable myocardium. Currently, we are not aware of and do not expect any influence of extracellular Pi on Ca^2+^ handling. Because cytosolic Pi is controlled through the Na^+^/Pi cotransporter Pit2 (Jobbagy et al., 1999), any elevation in cytosolic Pi would be expected to be accompanied by an elevation in cytosolic Na^+^, which would diminish sodium-calcium exchanger (NCX) activity and raise intracellular Ca^2+^ (Bers, 2002). This hypothetical scenario represents an attractive explanation for the enhanced systolic function with increasing extracellular Pi, but it would be contrary to the enhanced diastolic function also observed with extracellular Pi. We would also not attribute the Pi-enhancement of isometric contraction to an increase in calcium-sensitivity of the myofilaments, because Pi is known to reduce the calcium-sensitivity of myofilament force production (Kentish, 1986; Millar and Homsher, 1990).

We would instead attribute the Pi-enhancement of isometric contraction to the enhanced velocity of contraction that accompanies increasing Pi despite the reduction in crossbridge number (Tesi et al., 2000; Hinken and McDonald, 2004). The enhanced velocity of contraction with increasing Pi would have to make up for the loss of isometric force with increasing Pi. At this point, this is conjecture, and further studies into the mechanism underlying enhanced systolic function by extracellular Pi are warranted.

One final limitation to the current study is the lack of control for sarcomere length in the excitable myocardium. It is notoriously difficult to visualize and control for sarcomere length in myocardial preparations greater than 0.2 mm in diameter, but we would expect the sarcomere length of the excitable myocardium to be near the top of the force-length relatation between 2.1 and 2.3 μm (ter Keurs et al., 1980). In addition, the preparation was held isometric. Working myocardium would be expected to shorten during systole and sarcomere lengths at end-systole would be less than 1.9 μm (Gordon and Pollack, 1980). The effects of Pi on lengthening-induced relaxation, as we have examined in the current work, may be most relevant at these shorter sarcomere lengths.

## Conclusion

Our findings suggest that Pi enhances the stretch-dependent increase in cardiac myosin crossbridge detachment rate. Importantly, we found that this effect of Pi is most prominent with faster stretches, which is consistent with the findings of previously investigators (Chung et al., 2017). While these findings were observed by adjusting extracellular Pi, they are reflective of cytosolic Pi maintained by the Na^+^/Pi cotransporter Pit2, whose activity can be influenced by certain isoforms of protein kinase-C (Onwochei, 1995; Onwochei et al., 1998; Jobbagy et al., 1999). The modulation of cytosolic Pi *via* protein kinase control of Pit2 activity therefore represents a possible pathway for adjusting myocardial relaxation function.

## Data Availability

The raw data supporting the conclusion of this article will be made available by the authors, without undue reservation.
